# Haplotypes in SLC24A5 Gene as Ancestry Informative Markers in Different Populations

**DOI:** 10.2174/138920208784139528

**Published:** 2008-04

**Authors:** Emiliano Giardina, Ilenia Pietrangeli, Cristina Martínez-Labarga, Claudia Martone, Flavio de Angelis, Aldo Spinella, Gianfranco De Stefano, Olga Rickards, Giuseppe Novelli

**Affiliations:** 1 Centre of Excellence for Genomic Risk Assessment in Multifactorial and Complex Diseases, School of Medicine, Tor Vergata University of Rome, Italy; 2 Department of Biology, Tor Vergata University of Rome, Italy; 3 Centre of Molecular Anthropology for Ancient DNA Studies, Tor Vergata University of Rome, Italy; 4 Direzione Centrale Anticrimine, Servizio di Polizia Scientifica, Rome, Italy; 5 Division of Cardiovascular Medicine, Department of Medicine, University of Arkansas for Medical Sciences, Little Rock, AR 72205, USA

**Keywords:** SLC24A5, Ancestry Informative Markers (AIMs), forensic genetics.

## Abstract

Ancestry informative markers (AIMs) are human polymorphisms that exhibit substantially allele frequency differences among populations. These markers can be useful to provide information about ancestry of samples which may be useful in predicting a perpetrator’s ethnic origin to aid criminal investigations. Variations in human pigmentation are the most obvious phenotypes to distinguish individuals. It has been recently shown that the variation of a G in an A allele of the coding single-nucleotide polymorphism (SNP) rs1426654 within *SLC24A5* gene varies in frequency among several population samples according to skin pigmentation. Because of these observations, the *SLC24A5* locus has been evaluated as Ancestry Informative Region (AIR) by typing rs1426654 together with two additional intragenic markers (rs2555364 and rs16960620) in 471 unrelated individuals originating from three different continents (Africa, Asia and Europe). This study further supports the role of human *SLC24A5* gene in skin pigmentation suggesting that variations in *SLC24A5* haplotypes can correlate with human migration and ancestry. Furthermore, our data do reveal the utility of haplotype and combined unphased genotype analysis of *SLC24A5* in predicting ancestry and provide a good example of usefulness of genetic characterization of larger regions, in addition to single polymorphisms, as candidates for population-specific sweeps in the ancestral population.

## INTRODUCTION

The variation of pigmentation in humans is associated with melanosomes, the pigmented organelles of melanocytes, variable in number, size, and density [1]. Such variation is related to biological effects of UV radiation since dark skin offers more protection against UV irradiation at or near the equator, and pale skin protects people from vitamin D deficiency at higher latitudes. Although it has been hypothesized that many genes contribute to produce these different color shades, little is known about the biology and functions of these genes [2]. Lamason *et al*. (2005) showed that zebrafish 'golden' phenotype is due to mutations of the *SLC24A5* gene encoding for a protein belonging to the family of potassium-dependent sodium/calcium exchangers [3]. They observed that the G and A alleles of a coding single-nucleotide polymorphism (SNP) rs1426654, whose corresponding aminoacid is alanine or threonine, respectively, (amino acid position 111 in the third exon of *SLC24A5*; OMIM 609802. 0001), varies in frequency among several population samples according to skin pigmentation. For this specific feature, more recently, rs1426654 has been evaluated as Ancestry Informative Marker (AIM) together with the rs16891982 in the *SLC45A2 *gene [4]. An ancestry informative marker (AIM) is a human polymorphism that exhibits substantially different frequencies among populations. The data arising from single SNP approach demonstrated a non-full effectiveness of rs1426654 as ancestry informative marker because of its inability to well discriminate between Asians and other populations [4]. 

In order to improve the effectiveness of rs1426654 also in discrimination of Asian population we decided to type two new informative flanking SNPs (rs2555364 and rs16960620) other than the rs1426654 in 471 unrelated individuals originating from three different continents (Africa, Asia and Europe). 

It is well known that individuals who carry a particular SNP allele at one site often predictably carry specific alleles at other nearby varied sites. This correlation is known as linkage disequilibrium (LD). LD exists because of the shared ancestry of contemporary chromosomes. Early information documented that the human genome generally displays more LD rather than the one supposed for simple population genetic models, and that LD is more varied across genomic regions, being more segmentally structured rather than what had previously been supposed [5,6]. Thus there is a general agreement that individuals and populations can be differentiated each other by LD (haplotype) analysis better than single SNP approach. Therefore the human genome has specific regions showing large haplotype differences between populations; on this basis we liked to apply haplotype analysis of *SLC24A5* locus in order to verify the usefulness of typing specific and larger regions (which may be called Ancestry Informative Regions, AIRs) in addition to single SNP analysis in predicting ancestry of unknown samples. 

## MATERIALS AND METHODS

A total of 471 unrelated individuals originating from three different continents has been typed for rs2555364, rs1426654 and rs16960620. Selected individuals included Africans from Benin Gulf (172), Asians from Mongolia (115) and Europeans from Italy (184). The study protocol was approved by the ethical committee of “*Tor Vergata*” University in Rome. The QIAamp DNA Blood Mini Kit (QIAGEN Inc., Valencia, CA) was used to extract genomic DNA from whole blood. The SNPs rs2555364, rs1426654 and rs16960620, located within the *SLC24A5 *have been selected on the basis of allele frequencies reported in HapMap database (www.hapmap.org). The chromosomal locations of the three SNPs are reported in Table **1**. Genotyping of all the three SNPs was performed using the *Taq*Man allele discrimination assay (Applied Biosystems, Foster City, CA). 

The primers and probes sequences used for rs1426654, rs16960620 and rs2555364 were respectively: primers forward: 5’-GCCCTTGGATTGTCTCAGGAT-3’, 5’-AACCTTTCTAGCCCTGTAG-3’, 5’-CAGATAGCCCAGGTTCAAAT-3’, primers reverse: 5’-TGCCCGCTGCCATGAA-3’, 5’-TATTTCATTGCAGAGAGACTCAC-3’, 5’-CAGGAAAGATGAAA"GTGGAG-3’; probes 1: 5’-TTGCAGGCGCAACT-3’, 5’-TGTCTAAGTGACTTCCAACGTGCAC-3’, 5’-CTGTGTGACAAGTGACG-3’;.probes 2: 5’-TTGCAGGCACAACT-3’, 5’-AATATTGTTCATTAAGTGACTTAA-3’, 5’-CTGTGTGAGAAGTGACG-3’. 

Genotype assessment for rs2555364, rs1426654, rs1696-0620, has been confirmed by direct sequencing of random samples. Haplotypes were constructed separately in each populations by using the program PHASE [7]. No departures form Hardy-Weinberg equilibrium has been revealed for the three SNPs by using the program Arlequin. Haplotype frequencies were estimated using the Expectation-Maximization (EM) algorithm [8] implemented in the Arlequin v2.000 software, with number of iterations set at 5000, initial conditions set at 50, and an epsilon value of 10^-7^. Statistical independence of selected markers was assessed by calculating linkage disequilibrium (LD) as r^2^ [9] using LDplotter software (available at https://www.pharmgat.org/Tools/pbtold plotform). Inter-population variability in allele frequencies was assessed by calculating F_st_ for each marker (http://genepop.curtin.edu.au/genepop_op6.html). To test the null hypothesis of no differences in allele frequencies between HAPMAP and typed populations, the frequency distribution of alleles was analysed for our samples as well as for HAPMAP samples, using X^2^ test.

## RESULTS

Calculation of allele and haplotype frequencies for each SNPs are listed respectively in Tables **1-2**. Heterozygosity of the three SNPs is strongly decreased in Italian population, being only one chromosome carrying the 1 allele of the rs2555364. The other two SNPs were monomorphic in all Italian chromosomes tested. Heterozygosity of rs2555364 and rs1426654 is dissimilar in African population, with 68% of chromosomes carrying the 1 allele in the rs2555364 and 96% carrying the 2 allele of rs1426654. The rs16960620 is monomorphic in African as well as Italian samples, with all chromosomes carrying the 1 allele. More balanced allele frequencies have been revealed for all the three SNPs in Asian population, where 34% of chromosomes carries the 1 allele of rs2555364 and 64% the 2 allele of rs1426654. Only 10% of chromosomes carried the 2 allele of rs16960620. Haplotype analysis confirmed a more homogeneous chromosome pool in non- Asian populations where three different haplotypes (1-2-1 in Italian population; 1-1-2 and 1-2-2 in Benin population) describe the 99% and 96% respectively of chromosomal variability (Table **2**). The most of haplotypes analyzed exhibited dissimilar frequencies among different populations. Such differences in haplotype distribution can provide information about “biogeographical ancestry” and “demographic background” of unknown samples. For example, if an unknown sample carries the haplotype 1-1-2 it is likely it will not have an Italian ancestry and it has three times more probability to share an African ancestry versus an Asian. Furthermore, the haplotype 1-2-1 carried by the most of Italian chromosomes is present only in 3% of Benin chromosomes, and that the haplotype 1-1-2 and 1-2-2 carried by 66% and 30% respectively of Benin chromosomes were absent in Italian samples. Asian population showed a more heterogeneous chromosomes pool, in which 5 different haplotypes have a frequency of at least 3% (1-1-1; 1-1-2; 1-2-1; 2-1-2; 1-2-2). Only the haplotype 2-1-2 carried by 9% of chromosomes is exclusive of Asian population. In fact, 33% of Asian chromosomes have the “Italian” haplotype 1-2-1 and 32% have the haplotype 1-2-2 carried by 33% of Africans.

However it should be outlined that the reconstruction and the interpretation of haplotypes could be complicated for sporadic (single) samples and it is rarely applied in forensic analysis. To solve this matter we calculated the frequencies of combined genotypes without performing a phase reconstruction in each population (Table **3**). Combined unphased genotypes exhibited strongly dissimilar frequencies among different populations and suggest that this approach can be more useful to predict the ancestry of unknown samples. For example sample homozygous for the allele 1 at the rs169-60620, homozygous for the allele 2 at the rs2555364 and homozygous for the allele 1 at the rs1426654, on the basis of our frequency data (Table **3**) could unlikely have African ancestry and shows 9% and 91% of possibility to have Asian and Italian ancestry respectively. This analysis revealed 8 combined unphased genotypes selectively found in Asian populations representing the 17.2% of the total and able to discriminate between Asian and non-Asian populations.

These results were confirmed by calculating the *F*_st_ value (Table **4**). *F*_ST_ is the proportion of the total genetic variance contained in a subpopulation (the S subscript) relative to the total genetic variance (the *T* subscript). Values can range from 0 to 1. High *F*_st_ for specific markers implies a considerable degree of differentiation among populations. The higher the *F*_st_ value for a marker the higher its efficacy as a Ancestry Informative Markers. Combined unphased genotypes were able to better discriminate between Asian and non-Asian populations with respect to the single SNP rs1426654 as suggested by the *F*_st_ value. In particular combined unphased genotypes obtained a 21% of discrimination respect to 4% of rs1426654. 

## DISCUSSION AND CONCLUSION

The genetic investigation of diversity in human physical characters has a rich history and a long intrigued mankind. Here we report an investigation of haplotypes of *SLC24A5* as AIMs in providing individual ancestry estimates. This study further supports the role of human *SLC24A5* gene in skin pigmentation suggesting that variations in *SLC24A5* haplotypes can correlate with human migration and ancestry. The difference in haplotype frequencies between the major human groups (South Saharan African, Asian and European populations) is consistent with the possibility that this genomic region has been a target of natural or sexual selection. On the other hand, the evolution of the different skin colors may also have occurred as a consequence of adaptative selection for protection against pathogens (immunological selection for pigmentation).

Previous analysis of *SLC24A5* (rs1426654) revealed difficulties in discrimination between Asians and Africans [4]. By analyzing three SNPs within the *SLC24A5* we identified three haplotypes and eight different combined unphased genotypes exclusively observed in Asian populations (Tables **2-3**). *F*_st_ analysis further confirmed the utility of combined unphased genotypes of *SLC24A5* in predicting ancestry and therefore in providing further information on the physical characteristics of the individuals. Thus, the possibility of providing phenotypic information from a DNA profile of an evidence collected from a crime scene are becoming more and more helpful. However the biogeographical ancestry of an individual can be inferred with high reliability from genetic data if a much larger number of random markers is used, or specific “ancestry-informative” markers are chosen to have large allele frequency differential between population groups. Thus, in order to provide individual ancestry estimates with the lower associated standard errors and major precision the number of markers to analyze need to be really high. On the other hand, the study of the single genetic variations regarding the colour of the skin and other secondary physical characters which reflect adaptations of humans to the environment will help us to understand the physiological and biochemical processes of human variability. The availability of specific genetic databases can help us to detect those genes that have been positively selected in our divergence from chimpanzees, as well as those genes that have been under selection as human populations have migrated, differentiated, and adapted to specific geographic environments.

Variation in humans reflects genetic differences at single allele as well as haplotype level. At this respect, the HapMap project revealed 926 SNPs with allele frequencies that differ across the populations in a manner as extreme as the well-accepted example of selection at the Duffy (FY) locus. In addition 19 genomic regions (13 on autosomes, 6 on the X chromosome) has been identified as candidates for population-specific sweeps in the ancestral population [6,10]. Recently, the HapMap 2 analysis of recent positive selection revealed more than 300 candidate regions 22 of which were above a threshold such that no similar events were found in 10 Gb of simulated neutrally evolving sequence [6]. One of these 22 included the SLC24A5 gene. Thus, combined bioinformatics and genomic approach can significantly improve our ability to identify the genetic tags of ancestry easily selected *in silico* by using specific population databases and thereafter typed in large cohort of samples.

Although many efforts have been made first with STRs markers and then with SNPs [11,12] currently used AIMs are not 100% accurate for predicting ancestral background of samples especially for individuals with mixed ancestral background. Haplotype and especially combined unphased genotype analysis of *SLC24A5* here reported improved previous single allele results [4] and provided significant information about ancestry of samples which may be useful in predicting a perpetrator’s ethnic origin to aid criminal investigations. It is should be noticed that recent data suggest that the candidate regions for positive selection are large (mean length, 815 kb; maximum length, 3.5 Mb) and often contain multiple genes (median, 4; maximum, 15). A typical region harbours 400–4,000 common SNPs (minor allele frequency 5%), of which roughly three-quarters are represented in current SNP databases and half were genotyped as part of HapMap 2 [6]. In this perspective our possibility to predict the ancestry of unknown samples could be increased by studying AIRs in addition to AIMs.

## Figures and Tables

**Table 1.** Chromosomal Localization and Allele Frequencies of the SNPs in the European, African and Asian Populations

SNPs	Localization	Italians		Total	Africans		Total	Asians		Total
		Allele 1	Allele 2		Allele 1	Allele 2		Allele 1	Allele 2	
rs16960620	46,204,191	1	0	368	1	0	344	0,900	0,100	230
rs2555364	46,206,678	0,002	0,998	368	0,680	0,320	344	0,340	0,660	230
rs1426654	46,213,776	1	0	368	0,040	0,960	344	0,360	0,640	230

rs16960620 (allele A=1; allele G=2), rs2555364 (allele C=1; allele G=2), rs1426654 (allele A= 1; allele G=2).

**Table 2.** Haplotype Frequencies in the European, African and Asian Populations

Haplotype	Europeans	Africans	Asians
**111**	0,010	0,010	0.026
**112**	0	0,660	0,222
**211**	0	0	0,004
**121**	0,990	0,030	0,331
**221**	0	0	0,088
**122**	0	0,300	0,320
**222**	0	0	0,009

rs16960620 (allele A=1; allele G=2), rs2555364 (allele C=1; allele G=2), rs1426654 (allele A= 1; allele G=2).

**Table 3.** Frequencies of Combined Unphased Genotypes at rs16960620, rs2555364 and rs1426654. For Each Combined Unphased Genotype is Reported the Probability (in the Brackets) to be Carried by a People Originating from that Specific Population

	Europeans	Africans	Asians
**Ho_1_/Ho_2_/Ho_1_**	0,994 (0,91)	0	0,087 (0,09)
**Ho_1_/Ho_1_/He**	0	0,006 (0,23)	0,017 (0,77)
**Ho_1_/He/Ho_1_**	0,006 (0,16)	0,012 (0,31)	0,017 (0,53)
**Ho_1_/ He/He **	0	0,023 (0,08)	0,235 (0,92)
**He /Ho_1_/He**	0	0	0,017 (1,00)
**Ho_2_/Ho_2_/He**	0	0	0,009 (1,00)
**He/He/He**	0	0	0,078 (1,00)
**Ho_1_/He/Ho_2_**	0	0,407 (0,74)	0,139 (0,26)
**Ho_1_/Ho_1/_Ho_2_**	0	0,459 (0,94)	0,026 (0,06)
**Ho_1_/Ho_2_/Ho_2_**	0	0, 081 (0,34)	0,165 (0,66)
**Ho_1 _/Ho_2_/He**	0	0,012 (0,07)	0,139 (0,93)
**He /Ho_2_/Ho_2_**	0	0	0,009 (1,00)
**Ho_2 _/Ho_1_/Ho_2_**	0	0	0,009 (1,00)
**He /He/ Ho_2_**	0	0	0,035 (1,00)
**Ho_2_/Ho_1_/He**	0	0	0,009 (1,00)
**He/ Ho_1_/ Ho_2_**	0	0	0,009 (1,00)

Ho: homozigous; He:heterozygous. Alleles are indicated by subscripts number. rs16960620 (allele A=1; allele G=2), rs2555364 (allele C=1; allele G=2), rs1426654 (allele A= 1; allele G=2).

**Table 4.** Fst Values for both SNPs and Combined Unphased Genotypes

	Europeans *Vs* Africans	Europeans *Vs* Asians	Africans *Vs* Asians	Europeans *Vs* Africans + Asians	Africans *Vs* Europeans + Asians	Asians *Vs* Europeans + Africans
**rs2555364**	0,65	0,29	0,21	0,41	0,43	0
**rs1426654**	0,95	0,63	0,27	0,74	0,56	0,04
**rs16960620**	0	0,09	0,09	0,30	0,03	0,14
**TOTAL**	0,83	0,48	0,22	0,58	0,48	0,03
**combined unphased genotype**	0,75	0,62	0,29	0,56	0,46	0,21
